# Trends in Mortality and Risk Characteristics of Congenital Diaphragmatic Hernia Treated With Extracorporeal Membrane Oxygenation

**DOI:** 10.1097/MAT.0000000000000834

**Published:** 2019-05-23

**Authors:** Yigit S. Guner, Patrick T. Delaplain, Lishi Zhang, Matteo Di Nardo, Thomas V. Brogan, Yanjun Chen, John P. Cleary, Peter T Yu, Matthew T. Harting, Henri R. Ford, Danh V. Nguyen

**Affiliations:** From the * Division of Pediatric Surgery, Children’s Hospital of Orange County, Orange, California; † Department of Surgery, University of California Irvine Medical Center, Orange, California; ‡ Department of Surgery, University of California Irvine Medical Center, Orange, California; § Department of Pediatric Surgery, Children’s Hospital of Los Angeles, University of Southern California Los Angeles, CA; ¶ Institute for Clinical and Translational Science, University of California, Irvine, California; ‖ Pediatric Intensive Care Unit, Ospedale Pediatrico Bambino Gesu, Rome, Italy; # Department of Pediatrics, Seattle Children’s Hospital, University of Washington, Seattle, Washington; ** Division of Neonatalogy, Children’s Hospital of Orange County, Orange, California; †† Department of Pediatric Surgery, University of Texas McGovern Medical School, Houston, Texas; ‡‡ Department of Pediatric Surgery, Children’s Memorial Hermann Hospital, Houston, Texas; §§ Department of Medicine, University of California Irvine, Orange, California.

**Keywords:** ECMO, CDH, mortality risk, risk score, diaphragm, hernia

## Abstract

Although the mortality of infants with congenital diaphragmatic hernia (CDH) has been improving since the late 1990s, this observation has not been paralleled among the CDH cohort receiving extracorporeal membrane oxygenation (ECMO). We sought to elucidate why the mortality rate in the CDH-ECMO population has remained at approximately 50% despite consistent progress in the field by examining the baseline risk profile/characteristics of neonates with CDH before ECMO (pre-ECMO). Neonates with a diagnosis of CDH were identified in the Extracorporeal Life Support Organization (ELSO) Registry from 1992 to 2015. Individual pre-ECMO risk score (RS) for mortality was categorized to pre-ECMO risk-stratified cohorts. Temporal trends based on individual-level mortality by risk cohorts were assessed by logistic regression. We identified 6,696 neonates with CDH. The mortality rates during this time period were approximately 50%. The average baseline pre-ECMO RS increased during this period: mean increase of 0.35 (95% confidence interval [CI]: 0.324–0.380). In the low-risk cohort, the likelihood of mortality increased over time: each 5 year change was associated with a 7.3% increased likelihood of mortality (odds ratio [OR]: 1.0726; 95% CI: 1.0060–1.1437). For the moderate-risk cohort, the likelihood of mortality decreased by 7.05% (OR: 0.9295; 95% CI: 0.8822–0.9793). There was no change in the odds of mortality for the high-risk cohort (OR: 0.9650; 95% CI: 0.8915–1.0446). Although the overall mortality rate remained approximately constant over time, the individual likelihood of death has declined over time in the moderate-risk cohort, increased in the low-risk cohort, and remained unchanged in the high-risk cohort.

The mortality rate of infants with congenital diaphragmatic hernia (CDH) treated with extracorporeal membrane oxygenation (ECMO) has remained at 50% in the last 2 decades.^1–5^ Previous studies have not specifically evaluated the mortality risk profiles of neonates with CDH receiving ECMO. We hypothesize that the lack of improvement in gross mortality rate of neonates with CDH receiving ECMO may be secondary to changes in the risk profiles over time.

Although ECMO management is largely based on consensus protocols, ECMO initiation criteria vary among institutions with significant heterogeneity in practice patterns.^6^ The CDH-ECMO cohort represented in the ELSO Registry displays variation in practice patterns given the high number of centers that contribute data to ELSO. Variability in the application of ECMO and ECMO-specific outcomes are further coupled with the unmeasurable degree of unpredictability and chance for adverse outcomes given the complexity of the CDH disease state and of ECMO support. It is, therefore, imperative to adequately risk stratify a heterogeneous population, in an effort to study mortality risk trends and identify whether measurable changes in delivery of ECMO care have been made for the CDH-ECMO population. We are able to test the above-stated hypotheses as we have recently developed and validated mortality risk prediction equations specific for the CDH-ECMO population.^7^ In this study, we sought to 1) elucidate the baseline risk profile/characteristics of neonates with CDH before ECMO and 2) examine the trend in mortality for pre-ECMO risk-stratified cohorts over the last 2 decades.

## Methods

### Data Source and Cohort

This study was approved by the Children’s Hospital Orange County institutional review board (#150969). We queried the ELSO registry for neonates whose primary diagnosis was CDH from 1992 to 2015. ELSO made revisions/amendments to Registry data fields in 2016; we, therefore, limited the study period through the last case entry in 2015. An exhaustive search through all secondary International Classification of Diseases Ninth Revision, diagnosis codes was conducted to establish dichotomous variables to identify presence of comorbidities. The first ECMO run was used for each neonate. The final cohort included 6,696 neonates with CDH who were treated with ECMO. Mode of ECMO cannulation as venovenous (VV) or venoarterial (VA) were grouped as previously defined.^1^

### Pre–Extracorporeal Membrane Oxygenation Risk Strata

We used the individual pre-ECMO mortality risk score (RS) for the CDH population to define pre-ECMO risk strata (www.choc.org/ecmocalc).^7^ Using the individual pre-ECMO RS for mortality, neonates were recategorized into three pre-ECMO risk-stratified cohorts: low, RS ≤ 25% (RS < 0.5); moderate, RS 25–75% (RS 0.5–1.2); and high, RS > 75% (RS > 1.2), which were defined *a priori*. The RS includes information on weight before ECMO, Apgar at 5 minutes, side of hernia (left, right, and both), status of prenatal diagnosis of CDH, handbagging 6 hours before ECMO cannulation, whether the patient arrested before ECMO, whether diaphragmatic hernia was fixed (before ECMO), worst blood gas (pH) 6 hours before ECMO, pre-ECMO high frequency oscillatory ventilator (HFOV), concomitant diagnosis of CDH and critical congenital heart disease (CCHD),^8,9^ and presence of any perinatal infection. These were previously developed and validated from a list of 26 candidate patient risk factors.^7^

### Statistical Methods

Descriptive summary statistics, including mean and standard deviation (SD) or proportions for continuous and categorical baseline characteristics before ECMO (predictor variables of pre-ECMO RS) were provided for different periods: 1992–1999 (epoch 1); 2000–2009 (epoch 2); and 2010–2015 (epoch 3). Epochs were based on ending date of each decade and available data in Registry. The pre-ECMO RS in each decade was summarized as mean (±SD). Comparisons of patient characteristics between time periods were based on *t*-test and χ^2^ test for continuous and categorical variables, respectively. To examine the overall temporal trend in mortality, annual population mortality rate was estimated for each year using Poisson regression. Temporal trends in aggregate patient profile, defined by the continuous pre-ECMO RS, was examined using a linear regression model. Temporal trends in mortality among pre-ECMO risk-stratified cohorts were assessed by logistic regression. In post hoc analysis, we examined how post–gestational age and pre-ECMO CDH repair (effect modifiers) affect the trend in mortality over time by including interaction terms of year and effect modifiers in logistic regression models. Analyses were performed using SAS version 9.3 (SAS Institute Inc., 100 SAS Campus Drive, Cary, NC) and R version 3.2.2 (R-Studio, 250 Northern Ave, Boston, MA).

## Results

### Cohorts and Overall Mortality

We identified 6,696 neonates with CDH who were treated with ECMO from 1992 to 2015. The percentage of neonates in the low, moderate, and high pre-ECMO risk groups was similar in 2000–2009 and 2010–2015 (low: 28% vs. 23%; moderate: 57% vs. 50%; high: 25% vs. 27%; see **Table 1**). The number of neonates in each year ranged from 240 to 322, and the average number was 279 per year.

**Table 1.**
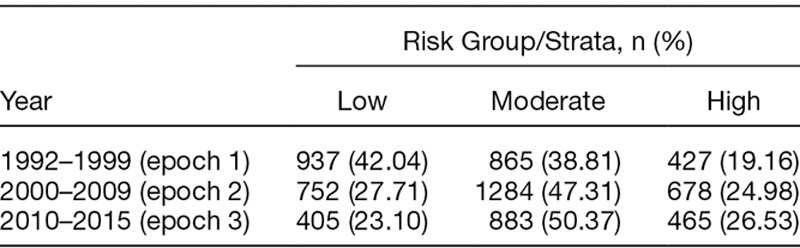
Frequency per Epoch

**Figure 1.**
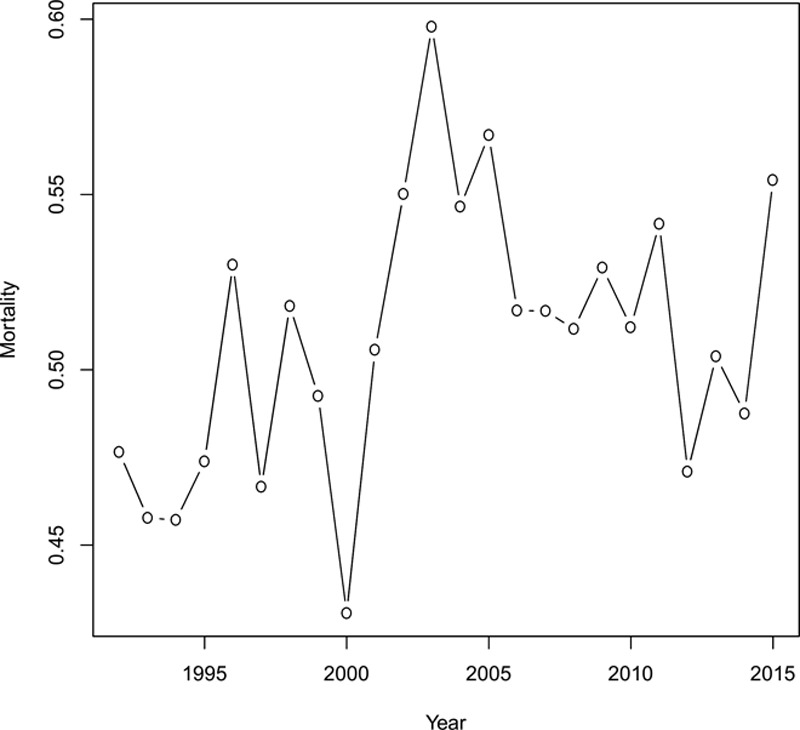
Annual mortality rates (1992–2015) with aggregate rate of 50% over this time period.

The annual unadjusted mortality rates during this period were stable at around 50% (ranging from 43.1% to 59.8%). The mortality rate in 1990s, 2000s, and 2010–2015 was 48.41%, 52.65%, and 51.17%, respectively. There was little evidence of overall changes in unadjusted mortality over time, from 1992 to 2015 (slope: 0.004; *p* = 0.1042; **Figure 1**).

### Baseline Pre–Extracorporeal Membrane Oxygenation Patient Risk Score Trajectory and Characteristics

**Figure 2.**
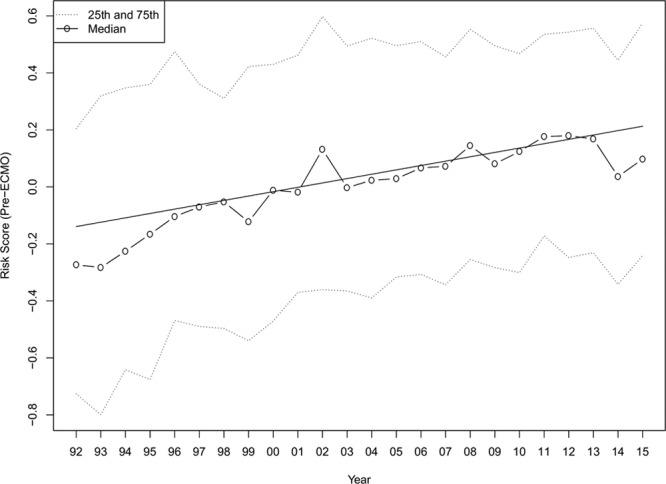
Baseline pre–extracorporeal membrane oxygenation (ECMO) risk score temporal trend (slope: 0.015; *p* < 0.0001). Solid line: model fit showing trend over time.

Although overall mortality during this period was stable at 50%, the average baseline pre-ECMO RS increased significantly during this period, specifically by 0.13% per year from 1992 to 2015 (slope: 0.015; 95% confidence interval [CI]: 0.014–0.016; *p* < 0.0001; **Figure 2**). Thus, the average increase in pre-ECMO RS trajectory from 1992 to 2015 was 0.35 units (95% CI: 0.324–0.380; *p* < 0.0001; **Figure 2**). This mean increase in RS of 0.35 over the last 2 decades represents 50% of the range of the RS in the moderate-risk group.

We next examined the individual pre-ECMO baseline characteristics in recent years (2010–2015 compared with the 1990s and 2000s). Overall, a higher prenatal diagnosis rate, lower pH and mean airway pressure (MAP), and higher prevalence of comorbidities including critical congenital heart disease, multiple congenital anomalies (MCA), and chromosomal anomalies were observed in recent years (**Table 2**). Also, age at ECMO initiation was higher in later years compared with earlier (**Table 2**). More specifically, the rate of prenatal diagnosis in 2010–2015 was 75.4%. It was significantly higher than 1990s (48.9%; *p* < 0.0001) and 2000s (60.3%; *p* < 0.0001). The mean pH value in 2010–2015 and 1990s was 7.13 (SD = 0.17) and 7.32 (SD = 0.19), respectively. We note that a decrease of 0.19 in pH corresponded to an increase of 0.28 in pre-ECMO RS. The mean MAP was 18.9 in 1990s and 16.06 in 2010s (*p* < 0.0001).

**Table 2.**
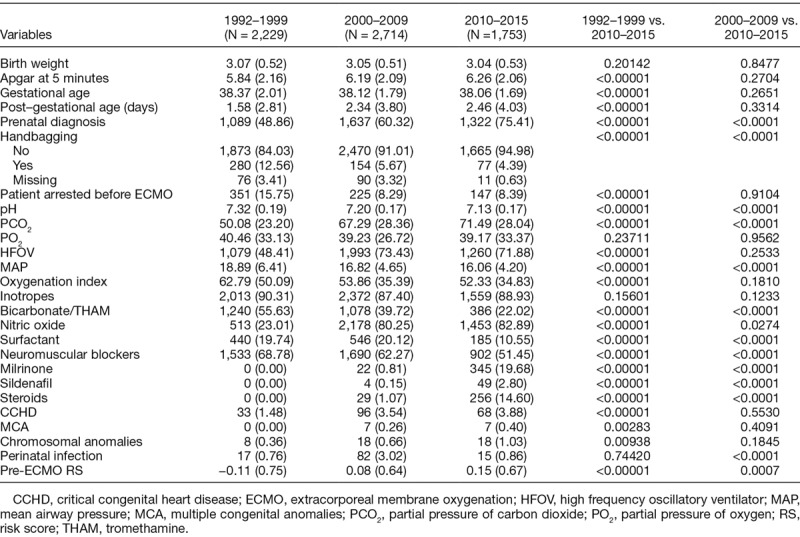
Pre-ECMO Variables per Epoch

The prevalence of CCHD in 2010s (3.8%) was significantly higher than 1990s (1.48%; *p* < 0.0001). Although extremely rare, 7 patients (0.4%) with MCA received ECMO in recent years, whereas no neonates diagnosed with MCA in 1990s received ECMO. The prevalence of chromosomal anomalies increased from 0.36% in 1990s to 1.03% in 2010s (*p* = 0.0094).

### Mortality Trajectories in Pre–Extracorporeal Membrane Oxygenation Risk Strata

We examined the temporal trend in mortality trajectories among the three pre-ECMO risk strata, defined *a priori* based on percentiles of the pre-ECMO RS. First, the percentage of patients in the low-risk group decreased from 42.04% to 23.10% in the recent 2 decades, whereas more neonates with moderate and high risk underwent ECMO (**Table 1**). The percentage of moderate and high group in 2010s increased 11.6% and 7.4% relative to 1990s, respectively.

**Figure 3.**
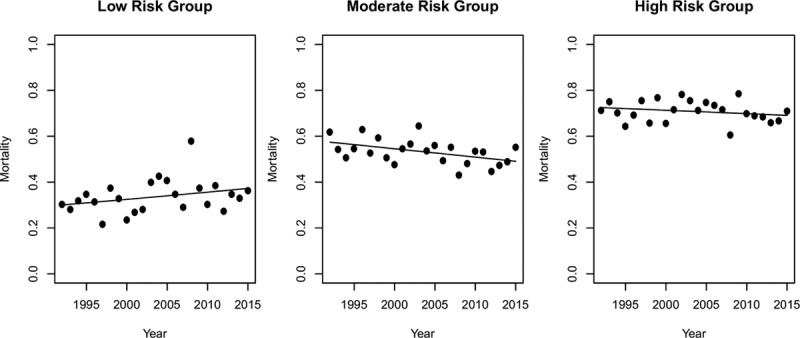
Individual-level model of mortality over years by *a priori* defined pre–extracorporeal membrane oxygenation (ECMO) risk cohorts.

We examined further, at the patient level, the odds (likelihood) of mortality over time within each of the 3 pre-ECMO risk cohorts (**Figure 3**). In the low-risk cohort, the likelihood of mortality increased over time: each 5 year change was associated with a 7.3% increased likelihood of mortality (odds ratio [OR]: 1.07; 95% CI: 1.006–1.143; *p* = 0.032). For the moderate-risk cohort, the likelihood of mortality decreased by 7.05% (OR: 0.93; 95% CI: 0.88–0.97; *p* = 0.006). There was no change in the odds of mortality for the high-risk cohort (OR: 0.96; 95% CI, 0.89–1.04; *p* = 0.37).

### Post Hoc Analysis of Modifier Effect on Mortality Trend

**Figure 4.**
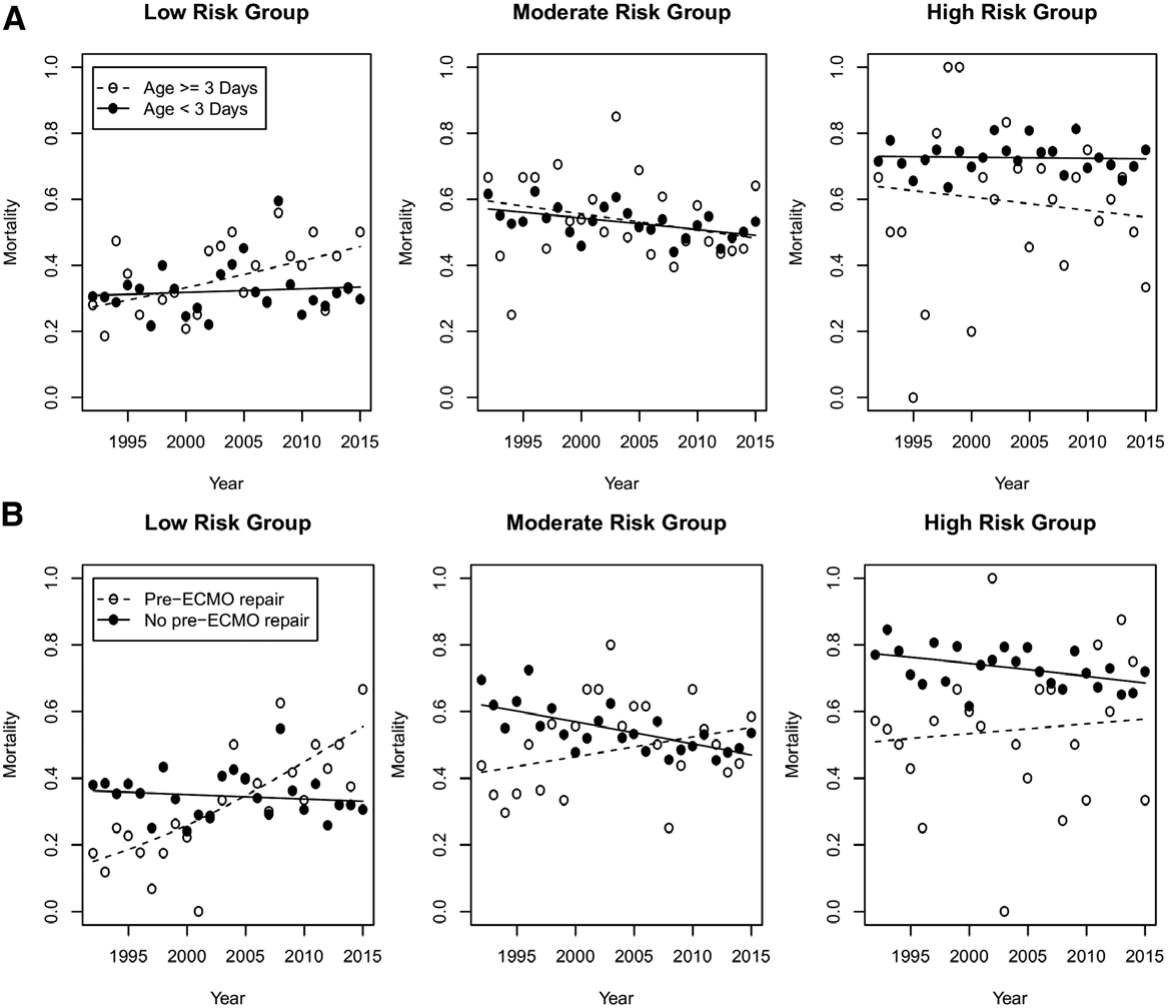
Individual-level model of mortality over years by (**A**) post–gestational age and (**B**) pre–extracorporeal membrane oxygenation (ECMO) congenital diaphragmatic hernia (CDH) repair groups in each *a priori* defined pre-ECMO risk cohort.

In post hoc analysis, we first examined whether post–gestational age and pre-ECMO CHD repair (effect modifiers) affect the trend of mortality over time. We found that post–gestational age and pre-ECMO repair were both significant in modifying the mortality trend over time in the low-risk group. In particular, increased mortality risk over time was attributed to neonates with age ≥3 days at cannulation (*p* = 0.006; **Figure 4**) in the low-risk group. Similarly, the mortality risk over time was attributed to neonates with pre-ECMO repair (*p* < 0.0001; **Figure 4**) among the low-risk group; there was no trend in mortality risk over time for low-risk neonates who did not have CDH repair before ECMO. Pre-ECMO CDH repair was also found to have a modifying effect on mortality trend over time in the moderate-risk group. However, unlike the low-risk group, moderate-risk neonates without pre-ECMO repair had a decreasing trend in mortality (*p* = 0.001; **Figure 4**), and no evidence of trend was found for the group with repair.

We also evaluated the effect of concomitant critical congenital heart disease. The number of infants with CCHD were 24 (1.1%) in low-risk group, 72 (2.4%) in moderate-risk group, and 103 (6.6%) in high-risk group. The mortality for high risk infants with CCHD is 72%, whereas the overall mortality in high-risk group was 71%. A sensitivity analysis was conducted for mortality trend in high-risk group by excluding those infants with CCHD. There was still no change in the odds of mortality for the high-risk cohort (5 year OR: 0.97; 95% CI: 0.89–1.05; *p* = 0.39).

### Trends in Extracorporeal Membrane Oxygenation Length, Mode, and Congenital Diaphragmatic Hernia Repair

The mean (SD) of length of ECMO for three epochs were 1.43 (0.87), 1.67 (1.02), and 1.70 (1.16) weeks for 1992–1999, 2000–2009, and 2010–2015, respectively. The duration of ECMO was significantly longer in 2010s compared with 1990s (*p* < 0.0001). However, no difference was detected between epochs 2 and 3. The frequency and percentage of use of VA cannulation is shown in **Table 3**. Venoarterial cannulation is more frequently used in 2010s compared with 2000s with approximate 4% increase. Epochs 1 and 3 were not found different in terms of modality. Approximately 43% infants were repaired on-ECMO for both 2000s and 2010s. The percentage of on-ECMO repair for 1990s is 5% more than the recent two epochs (**Table 3**).

**Table 3.**
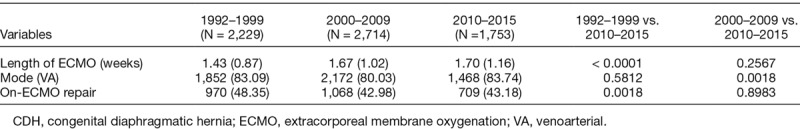
Trends in ECMO Length, Mode, and CDH Repair

## Discussion

As reported by the Congenital Diaphragmatic Hernia Study Group (CDHSG), the mortality of infants with CDH has been improving since the late 1990s.^10^ This observation, however, has not been paralleled among the CDH cohort receiving ECMO.^7,11^ In this study, we sought to specifically examine why the mortality rate in the CDH-ECMO population has remained at approximately 50% despite consistent progress in the field. We tested the hypothesis that lack of improvement in gross mortality rate could be because of worsening risk profiles in CDH patients over the course of the study period. We were able to carry out this study because, for the first time, we were able to calculate a validated, CDH-specific pre-ECMO mortality RS for each neonate.^7^ We then assigned the RSs to appropriate risk groups (low, moderate, and high), then studied the trends during study period to test the study hypothesis. Pre-ECMO characteristics of neonates in the later years of the study differed from the baseline profiles of neonates treated with ECMO in the 1990s. We showed that the pre-ECMO RS increased during the study period. In addition, mortality trends varied according to pre-ECMO characteristics. Mortality increased in the low-risk group, decreased in the moderate-risk cohort, but did not change in the high-risk group.

The average number of CDH-ECMO cases reported to the ELSO Registry annually remained stable during the study period. Even though the study denominator did not change, the subpopulations of different risk groups shifted toward higher risk groups comparing epoch 1 to epochs 2 and 3. Specifically, there was a decrease in the frequency of cases in the low-risk group, most likely because of advances in the management of neonatal respiratory distress, which have obviated the need for ECMO in the low-risk infants. There were statistically significant differences in the baseline pre-ECMO characteristics of infants between epoch 1 and 2. There were also differences between epochs 2 and 3, but far less in quantity of variables. It is interesting to note that the rate of prenatal diagnosis rose during the study period. Another interesting factor that is different among all three epochs of the study is increasing rate of handbagging and lower pre-ECMO pH, which may potentially point toward a preference toward late cannulation or increasing severity of patients. This is in contrast with the finding that patients were less likely to arrest in the later two epochs compared with epoch 1. Overall, the data from this observational study only present opportunities to identify areas for clinical improvement and identify additional research questions.

We next sought to understand differences within the CDH-specific pre-ECMO RSs of the different risk groups. Mortality over the study period as *a priori* defined by pre-ECMO risk cohorts demonstrated that pre-ECMO RSs increased for the low-risk group, decreased for the moderate-risk group, and remained unchanged for the high-risk group. We also know that even though the overall denominator has remained the same, the frequency of cases for low-risk infants is highest in epoch 1, lower in epoch 2, and even lower in epoch 3. This leads us to hypothesize that ECMO utilization rates may have decreased for the low-risk group given the recent advances in the management of neonatal respiratory distress. We further hypothesize that, in the later epochs of the study, ECMO utilization shifted toward the higher risk groups. A limitation of our study design is specific to the selection bias that is inherent to the ELSO registry, which is that ELSO only includes data on the population of infants born with CDH who were treated with ECMO. We cannot, therefore, make conclusions regarding ECMO utilization rate, as we do not know the true denominator. Future studies may be able to accurately study these possible shifts in ECMO utilization profiles from different data source, such as the CDH Study Group.

There are no additional studies with which we can compare the noted risk trends. The two-alternative means by which this cohort could be risk adjusted is by using either the Neonatal Risk Estimation Score in Children Using Extracorporeal Respiratory Support (Neo-RESCUER’s) or Pittsburgh Index for Pre-ECMO Risk (PIPER) RSs,^12,13^ which were developed for all neonates needing respiratory ECMO and were not specifically developed nor validated for the CDH population. Maul *et al*.,^13^ who developed PIPER, did describe a decline in overall survival for neonatal ECMO, without being specific for CDH. They further hypothesized that the increase in overall mortality observed for neonatal respiratory ECMO is a result of increase in severity illness. They, however, did not provide similar analyses to evaluate risk strata over the period of their study.

Our primary observations led to several post hoc analyses. Primarily, we asked whether age at ECMO cannulation, *i.e.*, the post–gestational age when infants were placed on ECMO, changed over time in the three different risk groups. When age was dichotomized to greater or less than 3 days of age, based on median age when ECMO cannulation occurred, we observed that in the low risk group mortality increased for cannulation at age ≥ 3 days. Furthermore, we asked whether there was a trend in cases where ECMO was needed after repair of CDH, and we noted that there was an increase in the number of pre-ECMO repairs over time in all risk groups but most prominently in the low-risk group. We, therefore, further hypothesize that the increase in mortality risk over time in the low-risk group is secondary to delay in age of cannulation and unplanned need for ECMO after CDH repair. There is evidence from CDHSG that the increasing use of inhaled nitric oxide (iNO) is associated with mortality.^14^ In our study, the use of iNO has increased from epoch 1 compared with epochs 2 and 3. It remains unclear what affect the use of iNO is having on delaying ECMO cannulation on infants who may otherwise go on to ECMO earlier without iNO. Milrinone use has also been increasing during the study period, and its effect on shifting trends of infants from low risk to high risk, by virtue to delaying ECMO cannulation, remains unknown. The milrinone in congenital diaphragmatic hernia trial^15^ may address these gaps in our understanding of the effect of pulmonary vasodilator therapy on CDH-related outcomes.

Limitations of our study include the retrospective nature in which the data were collected and analyzed, and the inherent selection bias of the ELSO Registry noted earlier. Overall ELSO error rate is believed to be near 1%.^16^ In identifying variables in the registry, we assumed that reporting in later years was the same as in earlier years, in assessing changes in presence of absence of predictor variables. It is possible that ECMO center volume or CDH center volume may play a role in outcomes measured in this study.^17–19^ The center identification is protected by the ELSO participation agreement and are not currently released for research purposes. We were, therefore, not able to test any specific hypothesis relating to center volume and timing of repair. Another key variable that would aid in prognostication would be the ability to measure trends not only in mortality risk but also in neurocognitive impairment rates, which is not possible with the ELSO registry data. Complications that occur during ECMO are not identified in the registry as the cause of mortality. Therefore, we were unable to link cause of mortality to trends in ECMO-related complications. Lastly, ELSO does not yet contain any quality metrics, with which we could apply to this dataset and compare trends in quality of care provided over time.

In conclusion, we have demonstrated specific changes within the different mortality risk profiles of infants with CDH treated with ECMO and challenged the notion that mortality rate has not changed in the last 2 decades. We have demonstrated that even though the gross mortality rate is near 50%, the risk profiles indicate that there were improvements in mortality risk profiles to the moderate-risk neonates. There was a decreasing frequency of low-risk infants placed on ECMO in later years but with an increasing mortality risk profile among those who were cannulated.
